# Push-Out Bond Strength of Restorations with Bulk-Fill, Flow, and Conventional Resin Composites

**DOI:** 10.1155/2015/452976

**Published:** 2015-09-20

**Authors:** Rodrigo Vieira Caixeta, Ricardo Danil Guiraldo, Edmilson Nobumitu Kaneshima, Aline Silvestre Barbosa, Cassiana Pedrotti Picolotto, Ana Eliza de Souza Lima, Alcides Gonini Júnior, Sandrine Bittencourt Berger

**Affiliations:** Department of Restorative Dentistry, School of Dentistry, University of North Parana (UNOPAR), Rua Marselha 183, 86041-140 Londrina, PR, Brazil

## Abstract

The aim of this study was to evaluate the bond strengths of composite restorations made with different filler amounts and resin composites that were photoactivated using a light-emitting diode (LED). Thirty bovine incisors were selected, and a conical cavity was prepared in the facial surface of each tooth. All preparations were etched with Scotchbond Etching Gel, the Adper Scotchbond Multipurpose Plus adhesive system was applied followed by photoactivation, and the cavities were filled with a single increment of Filtek Z350 XT, Filtek Z350 XT Flow, or bulk-fill X-tra fil resin composite (*n* = 10) followed by photoactivation. A push-out test to determine bond strength was conducted using a universal testing machine. Data (MPa) were submitted to Student's *t*-test at a 5% significance level. After the test, the fractured specimens were examined using an optical microscope under magnification (10x). Although all three composites demonstrated a high prevalence of adhesive failures, the bond strength values of the different resin composites photoactivated by LED showed that the X-tra fil resin composite had a lower bond strength than the Filtek Z350 XT and Filtek Z350 XT Flow resin composites.

## 1. Introduction

The contraction of dental composites is reported to be approximately 1–5% of their volume [1, 2]. The insertion of these contracting materials into bonded preparations induces the development of mechanical stress inside the material [2]. The stress is then transmitted via the bonded interfaces to the tooth structure [2, 3]. In a totally elastic situation, according to Hooke's law, stress should be determined by the product of the volumetric shrinkage and the elastic modulus (E-modulus) of the material [4]. Though the setting of a dental composite is not a purely elastic situation, an increased E-modulus has been related to higher stress [4–6] and the higher the E-modulus, the greater the stiffness. Thus, in light-cured composites, a rapid conversion induces a correspondingly rapid increase in composite stiffness, causing high shrinkage stresses at the restoration-tooth interface. Such stresses may disrupt the bonding between the composite and the cavity walls or may even cause cohesive failure of the restorative material or the adjacent tooth tissue [2].

Leakage or microleakage occurs in conjunction with all dental restorations and has been defined as the “clinically undetectable passage of bacteria, fluids, molecules, or ions between a cavity wall and the restorative material applied to it” [7–9]. Debonding occurs at the interface when the shrinkage stress exceeds the bond strength [10]. As a result, a number of problems may arise, such as precipitating clinical and radiographic sequelae including marginal staining and microgap formation (approximately 10 to 20 *μ*m), hypersensitivity, secondary caries, pulp inflammation, and, finally, restoration removal and reinsertion as final outcomes [9, 11–13].

Reducing the amount of polymerization shrinkage is an important issue in the development of dental resin composites. Nonshrinking resins and modified filler particles have been developed to tackle this problem, but they have not yet been shown to clinically last [6, 14]. Factors that can affect shrinkage are inorganic filler content, the molecular weight of the monomer system, and the degree of conversion of the monomer system [6, 14]. Previous studies have shown that approximately 90% of the shrinkage occurs within the first hours of polymerization [6, 15]. During setting of the resin composites, polymerization shrinkage induces contraction stress [6, 16]. While during curing, not all of the shrinkage is converted to contraction stress because the polymer can rearrange and relieve stress. In principle, this flow is composed of a macroscopic and microscopic component. Macroscopic flow occurs at the free surfaces during the polymerization reaction, which is evident by the development of a meniscus on the latter surfaces [6, 17]. Microscopic flow is due to polymer rearrangement within the resin composite. The molecular structure, crosslink density of the network, interaction of the matrix and filler particles, and reaction kinetics may play a role in this type of flow [6].

Due to considerable improvements since their inception, the use of photopolymerizable resin-based composite restorative materials has been more frequently extended to large and deep cavities, albeit with variable success [18, 19]. In such cases, the incremental build-up of multiple thin layers is required because of the limited cure depth [19, 20] and to potentially reduce the consequences of shrinkage stress [19, 21], although the latter theory has been refuted [19, 22]. However, layering techniques and multiple curing regimens of resin composites are time consuming [19]. Consequently, the composite material market is often driven by consumer demand for faster and easier procedures (sometimes at the cost of fundamental materials science principles) that reduce the curing time and/or use thicker composite layers. For example, “bulk-fill” materials, which are claimed to enable restoration build-up in thick layers, up to 4 mm, have become increasingly popular among dental practitioners [19]. Thus, the purpose of this study was to evaluate the bond strengths of composite restorations photoactivated using a light-emitting diode (LED) that were made with different filler amounts and resin composites. The null hypothesis tested was that there is no difference in bond strength among the composite restorations photoactivated using LED that were made with different filler amounts and resin composites (bulk-fill, flow, and conventional resin composites).

## 2. Materials and Methods

**Figure 1:**
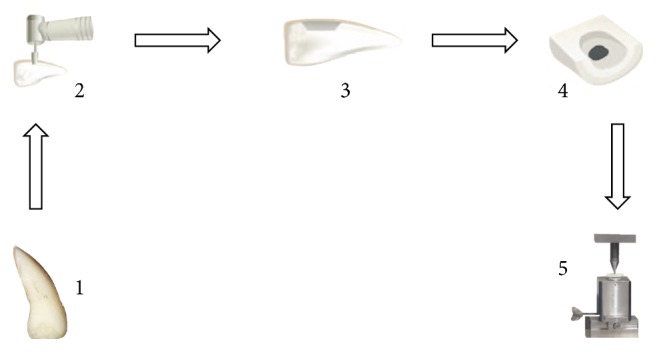
Schematic representation of the “push-out” test: (1) bovine incisor; (2) cavity preparation using standard cavity preparation appliance; (3) lateral view of the restored sample; (4) selective wear of the lingual surface and exposure of the bottom area of the restoration; (5) lateral view of the testing setup.

Thirty bovine incisors were selected (Figure 1(1)), and the crowns were cut off at the cementoenamel junction with a double-faced diamond disk (KG Sorensen, São Paulo, Brazil). The buccal surfaces of the crowns were wet-ground using an automatic polisher using 600-grit SiC sandpaper.

A conical cavity (top diameter of 4.5 mm, bottom diameter of 4.0 mm, and height of 2.5 mm) was prepared in the buccal surface of each tooth using a #3131 diamond tip (KG Sorensen) using a high-speed handpiece with a copious air-water spray and using a standard cavity preparation device (Figure 1(2)). The diamond tip was replaced after every five preparations. The cavity configuration factor (C-factor) was approximately 3.0.

All preparations were etched with Scotchbond Etching Gel, containing 35% phosphoric acid (Batch number N384021; 3M ESPE, St. Paul, USA), for 30 s for enamel and 15 s for dentin and washed with water for 30 s; the excess moisture was removed with absorbent paper. The Primer (Batch number N198771; 3M ESPE) and the Adhesive Adper Scotchbond Multipurpose Plus (Batch number N335871; 3M ESPE) were applied to the preparations according to the manufacturer's instructions and photoactivated using a LED curing unit (Radii Cal, SDI, Bayswater, Victoria, Australia) with an irradiance of 1400 mW/cm^2^ and an exposure of 20 s. Thereafter, the conventional nanoparticle-filled composite Filtek Z350 XT (Batch number 775639; 3M ESPE), the conventional nanoparticle-filled composite Filtek Z350 XT Flow (Batch number N509855; 3M ESPE), or the bulk-fill X-tra fil composite (Batch number 1315355; Voco, Cuxhaven, Germany), (Table 1) was inserted into the cavity in a single increment (Figure 1(3)) and photoactivated using a LED for 40 s (*n* = 10).

After light curing, the specimens were stored in distilled water at 37°C for 24 hours and then finished with Sof-Lex (3M ESPE). Then a 3017HL diamond tip (KG Sorensen) was used to ground the lingual face of the crown in order to expose the bottom surface of the restoration. The mesial and distal areas of the crown in the lingual surface were preserved as a mode of reinforcing the specimen for the push-out test (Figure 1(4)).

The push-out test was performed to evaluate the bond strength. An acrylic device with a central hole was adapted on the base of a universal testing machine (EMIC DL2000, EMIC Equipamentos e Sistemas de Ensaio Ltda., São José dos Pinhais, Brazil). The central hole was used for positioning the specimen with its cavity bottom side up (smaller diameter of the restoration cavity). In the superior area of the machine, a round tip was adapted (Figure 1(5)). This tip applied a compressive force on the bottom surface of the restoration in order to provoke the rupture of the tooth-composite bond along the lateral walls. The speed used in the test was 0.5 mm/min.

The values recorded (kgf) were divided by the area bonded and converted into pressure values (MPa). Statistical analysis was performed with the Minitab 16 program for Windows 8 (Minitab, State College, PA, USA). Normality of the data distributions was investigated by the Kolmogorov-Smirnov normality test. Subsequently, parametric tests were used. Data for the bond strength were submitted to Student's *t*-test at a 5% significance level. After the test, the fractured specimens were examined using an optical microscope (SZM; Bel Engineering srl, Monza, Italy) under magnification (10x), and the modes of failure were classified as follows: adhesive failure, cohesive failure within the composite, or mixed failure involving adhesive, dentin, and composite.

## 3. Results

The bond strength results are shown in Table 2. The bond strength values of the different resin composites showed that the X-tra fil (5.12 ± 1.21) resin composite had a lower bond strength than the Filtek Z350 XT (6.54 ± 0.94) and Filtek Z350 XT Flow (6.76 ± 1.53) resin composites (*p* = 0.014).

**Figure 2:**
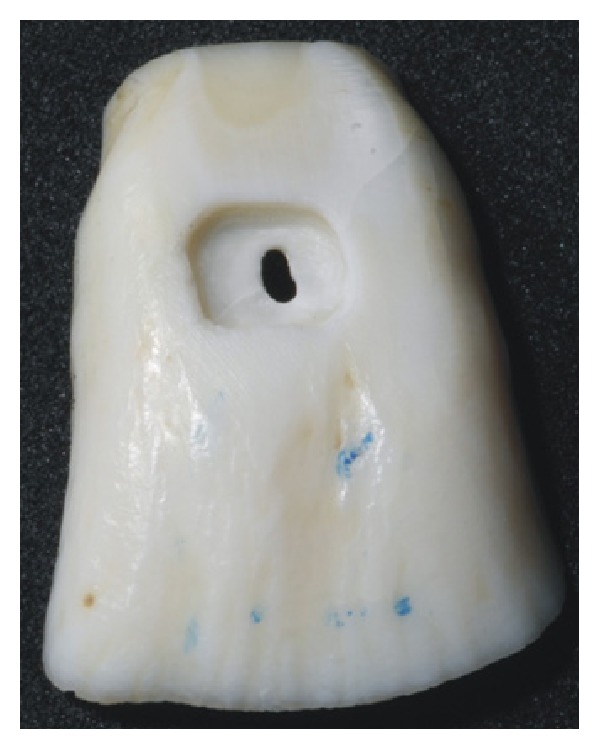
Illustration of adhesive failure mode.

**Figure 3:**
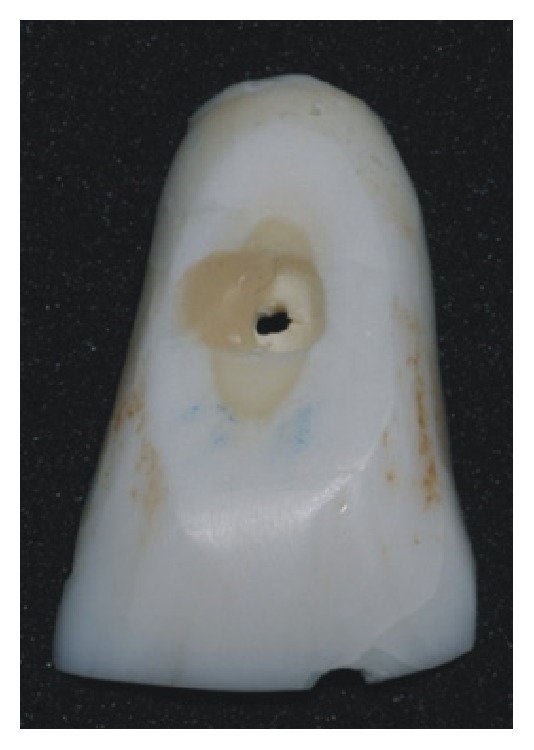
Illustration of mixed failure mode.

Classification of the modes of failure for the three composite restorations with different filler amounts and resin composites is shown in Table 3. The three composites demonstrated a high prevalence of adhesive failures (X-tra fil, 90%; Filtek Z350 XT, 80%; and Filtek Z350 XT Flow, 80%).  Figure 2 illustrates adhesive failure mode and Figure 3 illustrates mixed failure mode.

## 4. Discussion

Usually, the push-out test is used to evaluate the bond strength of endodontic cements to the radicular conduit [2, 23]. However, in the present study, the push-out test was adapted to evaluate the bond strength of restorative composites in a simulated Class V cavity as described in another study [2] that examined the influence of irradiation on restorative composites. Other bond strength tests such as shear, tensile, microshear, and microtensile evaluations are usually carried out to evaluate the bond strength of resin composites [2]. However, these tests are generally performed on flat surfaces [2]. In such a situation, the C-factor (the cavity configuration factor is the ratio of the bonded surface area to unbounded or free surface area) is very low and the development of shrinkage stress is not directed toward the bonding interface [2]. The advantage of using the push-out test is its ability to evaluate bond strength in a high C-factor cavity (3.0) with high stress generation directed toward the bonding area [2]. Thus, in the present study, the entire bonding area was submitted to the compressive force at the same time, allowing the shear bond strength to be evaluated in a cavity. In addition, the confidence of the push-out test was confirmed by the low data variability and low standard deviations. Furthermore, analysis of the mode of failure showed a high prevalence of adhesive failure for all the composites tested.

Currently, there is a growing trend among practitioners to use bulk-fill materials because of their more simplified procedures [19]. However, the lack of available literature on their clinical performance has stimulated much* in vitro* research, which ranks the properties of bulk-fill materials relative to the conventional flow and paste composite types already on the market. In the available literature, some interesting characteristics have been reported for bulk-fill materials [19]. First, the possibility of adequately light-curing these materials to greater than 4 mm thickness was confirmed by microhardness measurements for X-tra fil (VOCO) [19, 24]. However, the use of such methods to assess the cure quality may lead to an overestimation of the cure depth [19, 20]. Moreover, the extent of the cure depth indirectly evaluated by biaxial flexural strength measurements was significantly lower (<4 mm) than when relying on the degree of conversion or microhardness measurements [19, 25]. In the present study, the restorations made with the X-tra fil resin composite showed lower mean bond strength values than those achieved with the Filtek Z350 XT and Filtek Z350 XT Flow resin composites.

These results can probably be explained by the existence of differences in the composition of the materials studied. In X-tra fil, the organic matrix is composed mainly of bisphenol A glycidyl methacrylate (bis-GMA), urethane dimethacrylate (UDMA), and triethylene glycol-dimethacrylate (TEGDMA), while its inorganic particles comprise 70.1% of the volume. In contrast, in Filtek Z350 XT, the organic matrix is composed of bis-GMA, UDMA, TEGDMA, and ethoxylated bisphenol A-methacrylate (bis-EMA), and its inorganic components (63.3% of the volume) are silica (20 nm), zirconia (4–11 nm), and zirconia/silica clusters (0.6–10 *μ*m). Meanwhile, Filtek Z350 XT Flow is composed of bis-GMA, TEGDMA, and Procrylat K (replacement dimethacrylate), and its inorganic particles (46% of the volume) are yttrium fluoride (0.1–5.0 *μ*m), silica (20 nm), zirconia (4–11 nm), and zirconia/silica clusters (0.6–10 *μ*m). The lower mean bond strength obtained for X-tra fil may be explained by differences in the organic matrix composition among the materials. The main contributing factors to the reduced shrinkage of bulk-fill materials are their low flexural modulus and low filler loading [22]. However, according to Leprince et al. [19], X-tra fil has features similar to microhybrid restorative materials with high filler loading (according to the manufacturers, Table 1), possibly explaining our findings.

Resin composites exhibit viscoelastic behavior and are transformed during polymerization from a viscous plastic to a rigid elastic structure [1, 26, 27]. The polymerization shrinkage of the matrix, combined with a limited adhesion force of adhesive systems to dental tissue, challenges the stability of a restoration [27, 28]. In addition, adhesive bonding of composites to teeth results in contraction stresses, the magnitude of which is dependent upon several factors [27]. Thus, the development of contraction stress in dental composites depends upon the material composition, including the type of monomer; the type and amount of filler; filler/matrix interactions; polymerization parameters such as the degree and rate of polymerization; placement; and curing technique [4, 27]. In this study, the two composite resins Filtek Z350 XT and Filtek Z350 XT Flow had similar bond strengths, possibly because these factors were balanced. Based on the results of this study, the null hypothesis must be rejected because there was a difference in bond strength among the composite restorations photoactivated using LED that were made with different filler amounts and resin composites (bulk-fill, flow, and conventional resin composites).

## 5. Conclusions

Based on the results of this study, the X-tra fil composite restoration system resulted in a push-out bond strength less than those of Filtek Z350 XT and Filtek Z350 XT Flow composite restorations. Thus, the X-tra fil composite restoration system would show lower values in a Class V cavity when compared to other composites used in this study.

## Figures and Tables

**Table 1:** Information about the composites employed according to the manufacturer.

Composite	Organic matrix	Filler	Shade	Batch number
Filtek Z350 XT	bis-GMA, UDMA, TEGDMA, and bis-EMA	63.3% of the volume (silica: 20 nm, zirconia: 4 to 11 nm, and zirconia/silica clusters of 0.6 to 10 *μ*m)	A2B	775639

Filtek Z350 XT Flow	bis-GMA, TEGDMA, and Procrylat K	46% of the volume (yttrium fluoride: 0.1 to 5.0 *μ*m, silica: 20 nm, zirconia: 4 to 11 nm, and zirconia/silica clusters of 0.6 to 10 *μ*m)	A2	N509855

X-tra fil	bis-GMA, UDMA, and TEGDMA	70,1% by volume (inorganic fillers)	U	1315355

**Table 2:** Mean of push-out bond strengths (MPa).

Composite	Bond strengths (MPa)
Filtek Z350 XT	6.54 (0.94)^a^
Filtek Z350 XT Flow	6.76 (1.53)^a^
X-tra fil	5.12 (1.21)^b^

Mean values followed by different lowercased letters in the column differed statistically by Student's *t*-test at 5% level for different composites. Standard deviations are given in parentheses.

**Table 3:** Percentage (%) of failure mode.

Composite	Failure mode		
	Cohesive	Adhesive	Mixed
Filtek Z350 XT	0 (0)	80 (8)	20 (2)
Filtek Z350 XT Flow	0 (0)	80 (8)	20 (2)
X-tra fil	0 (0)	90 (9)	10 (1)

The modes of failure were classified as follows: adhesive failure, cohesive failure within the composite, or mixed failure involving adhesive, dentin, and composite. The number of the specimens is given in parentheses.
